# Development and validation of a nomogram model for assessing the severity of acute pancreatitis from the perspective of PICS

**DOI:** 10.3389/fnut.2025.1611501

**Published:** 2025-10-09

**Authors:** Dongquan Liu, Hongfei Liu, Jiaming Yao, Gan Chen, Weichen Wang, Baoqiang Cao, Jinlong Hu, Wenyong Wu

**Affiliations:** ^1^Anhui No.2 Provincial People’s Hospital Clinical College, Anhui Medical University, Hefei, China; ^2^Department of General Surgery, Anhui No.2 Provincial People’s Hospital, Hefei, China; ^3^Graduate School, Bengbu Medical University, Bengbu, China

**Keywords:** acute pancreatitis, persistent inflammatory–immunosuppressed–catabolic syndrome, nutrients consumption, systemic immune–inflammation index, neutrophil lymphocyte ratio

## Abstract

**Background:**

Early and convenient prediction of the severity of acute pancreatitis (AP) is crucial for its treatment and prognosis. This study aimed to develop and validate a nomogram model for assessing the risk of severe acute pancreatitis (SAP) based on the theory of Persistent Inflammation, Immunosuppression, and Catabolism Syndrome (PICS).

**Methods:**

A total of 4,930 AP patients from the MIMIC-IV database were selected as the derivation cohort, which was divided into the SAP group (*n* = 975) and non-severe acute pancreatitis (NSAP) group (*n* = 3,955) according to the 2012 Atlanta classification criteria. The 9 hematological indicators collected at the earliest time point within 48–72 h of admission were subjected to logistic regression analysis, and the statistically significant indicators selected were used to establish the model. A validation cohort consisting of 233 AP patients (34 in the SAP group and 199 in the NSAP group) admitted to the Department of General Surgery, Anhui No.2 Provincial People’s Hospital from January 2016 to October 2024 was used to verify the model’s performance.

**Results:**

Multivariate Logistic regression showed that neutrophil-lymphocyte ratio (NLR), systemic immune-inflammation index (SII), white blood cell count (WBC), hemoglobin (Hb), and red blood cell distribution width (RDW) were independent predictors of SAP (*P* < 0.05). The nomogram model equation was constructed as follows: logit(P) = ln(2.37)⋅ log(NLR) + ln(0.45)⋅ log(SII) + ln(2.60)⋅ log(WBC) + ln(0.85)⋅ Hb + ln(1.14)⋅ RDW. The area under the receiver operating characteristic curve (AUC) of the derivation cohort was 0.730 (95% CI: 0.708–0.743), with a Hosmer-Lemeshow test *P*-value of 0.333. The AUC of the validation cohort was 0.795 (95% CI: 0.703–0.886).

**Conclusion:**

The nomogram model based on NLR, SII, WBC, Hb, and RDW has good predictive value for SAP and can provide a convenient tool for early clinical identification of SAP.

## 1 Introduction

Acute Pancreatitis (AP) is a common clinical acute abdomen with significant differences in severity. Non-severe acute pancreatitis (NSAP) is mostly self-limiting, while severe acute pancreatitis (SAP) is associated with a mortality rate of up to 20% due to the vicious cycle of persistent inflammation, immunosuppression, and catabolism (1). The 48–72 h after admission is a critical window for AP progression: during this period, fluid imbalance in patients is basically corrected, making hematological indicators more valuable for reference; meanwhile, the systemic inflammatory response syndrome (SIRS) reaches its peak and gradually transitions to Persistent Inflammation, Immunosuppression, and Catabolism Syndrome (PICS) (2, 3). PICS is a clinical syndrome characterized by persistent inflammatory response, immunosuppression, and high protein catabolism, which is the core pathophysiological feature of SAP (4, 5). Compared with ordinary AP, SAP not only has persistent inflammation or infection but also is accompanied by inability to eat, massive nutritional consumption, and catabolism, resulting in higher mortality (1). Thus, early identification of PICS is of great significance for the treatment of SAP patients. However, the clinical diagnostic criteria for PICS are complex and cumbersome, and not yet unified, which limits its clinical application to a certain extent (6). Currently, commonly used scoring systems such as BISAP, Ranson, MCTSI, and APACHE II cannot well reflect this process, and are complicated to operate with certain lag (7). Based on the above, we aim to find a simple evaluation method that can simultaneously reflect inflammatory response and nutritional consumption to help clinicians detect patients with PICS as early as possible to prevent progression to SAP.

Complete Blood Count (CBC) is an easily accessible, inexpensive, and rapid test. The increase in WBC is mainly driven by a sharp increase in neutrophils, which is the most direct and classic sign of persistent inflammation (8). Neutrophil-Lymphocyte Ratio (NLR) is a golden indicator reflecting both inflammation and immune status. The increase in NLR perfectly captures the core contradiction that the inflammatory storm continues but the immune system has begun to collapse, making it a strong predictor of poor prognosis in SAP patients, thus being widely discussed (9). Systemic Immune-Inflammation Index (SII) (platelet count × neutrophil count / lymphocyte count) is a new marker that incorporates platelets on the basis of NLR, further reflecting the association between coagulation disorders and immunosuppression (10). Hb and RDW reflect catabolism and inflammatory nutritional consumption (11, 12).

Therefore, monitoring these indicators during the important early window of AP patients not only helps judge the severity of the disease but also, more importantly, alerts clinicians to the need for early intervention: while actively controlling inflammation (such as fluid resuscitation and organ support), attention must be paid to immune regulation and nutritional support (such as early enteral nutrition and possible immunomodulator research) as early as possible to try to break the vicious cycle of PICS and prevent the development of SAP.

## 2 Materials and methods

### 2.1 Study subjects

#### 2.1.1 Derivation cohort

This was a retrospective cohort study. Data for the derivation cohort were obtained from the clinical records of 4,930 AP patients (with ICD-10 primary diagnosis of “acute pancreatitis”) from the MIMIC-IV 3.1 database (2008–2022). Missing values were handled using the random forest imputation method (13) (20 iterations were set, generating 5 imputed datasets). The Rubin’s rules were used to pool the analysis results. According to the 2012 revised Atlanta classification criteria (14), patients with persistent organ failure for more than 48 h were defined as SAP (*n* = 975), and the rest were NSAP (*n* = 3,955). Organ failure was determined based on a SOFA score ≥2 points (respiratory failure: PaO_2_/FiO_2_ ≤ 300; circulatory failure: systolic blood pressure <90 mmHg requiring vasoactive drugs; renal failure: creatinine >171 μmol/L or urine output <0.5 ml/kg/h) (15).

#### 2.1.2 Validation cohort

A total of 233 AP patients admitted to the Department of General Surgery, the Anhui No.2 Provincial People’s Hospital from January 2016 to October 2024 were included. AP was diagnosed if any 2 of the following 3 criteria were met (14): (1) characteristic abdominal pain of AP; (2) serum amylase and/or lipase ≥3 times the upper normal limit; (3) characteristic CT findings of AP. Patients were divided into the SAP group (*n* = 34) and NSAP group (*n* = 199) according to the same criteria. The same hematological indicators were collected.

Inclusion criteria: 1. Meets the diagnosis of AP. 2. Hospital stay ≥48 h. 3. Relatively complete data.

Exclusion criteria: (1) Patients who underwent surgery during hospitalization; (2) Patients who received blood transfusion or albumin transfusion during hospitalization; (3) Patients with hematological diseases; (4) Patients with active bleeding; (5) Patients with cancer, tumors, or autoimmune diseases; (6) Diseases that may interfere with blood indicators, such as chronic liver disease and end-stage renal disease; (7) Recent history of chemotherapy.

### 2.2 Data collection

Hematological indicators collected at the earliest time point within 48–72 h of admission included white blood cells (WBC), neutrophils (N), lymphocytes (L), hemoglobin (Hb), red blood cell distribution width (RDW), platelets (PLT), and albumin (Alb). CRP and PCT were not collected due to high missing rates. Neutrophil-lymphocyte ratio (NLR) = N/L and systemic immune-inflammation index (SII) = PLT*NLR were calculated.

### 2.3 Ethics and informed consent

This study obtained the following ethical approvals:

#### 2.3.1 Derivation cohort

Data were obtained from the MIMIC-IV 3.1 database after obtaining the necessary access permissions through the official application process (CITI PROGRAM Record ID: 71643826), in compliance with database usage regulations (16).

#### 2.3.2 Validation cohort

Approved by the Ethics Committee of the Anhui No.2 Provincial People’s Hospital (Ethics No.: (R) 2025-023). Since this was a retrospective study with all data anonymized, the Ethics Committee approved the waiver of informed consent, in accordance with the Declaration of Helsinki.

### 2.4 Statistical analysis

Multivariate Logistic regression analysis was used to identify factors influencing the progression of AP to SAP. Analyses were performed using R software (version 4.3.0) and the rms package. The discriminative ability and calibration plots of the validation set were used to evaluate the accuracy of the nomogram. Receiver operating characteristic (ROC) curves were plotted and the area under the curve (AUC) was calculated to evaluate the discriminative ability of the nomogram. The Hosmer-Lemeshow test was used to verify model consistency, and decision curve analysis (DCA) was plotted to evaluate the model’s discriminative ability. A *P*-value < 0.05 was considered statistically significant.

## 3 Results

### 3.1 Data imputation and validation

**FIGURE 1:**
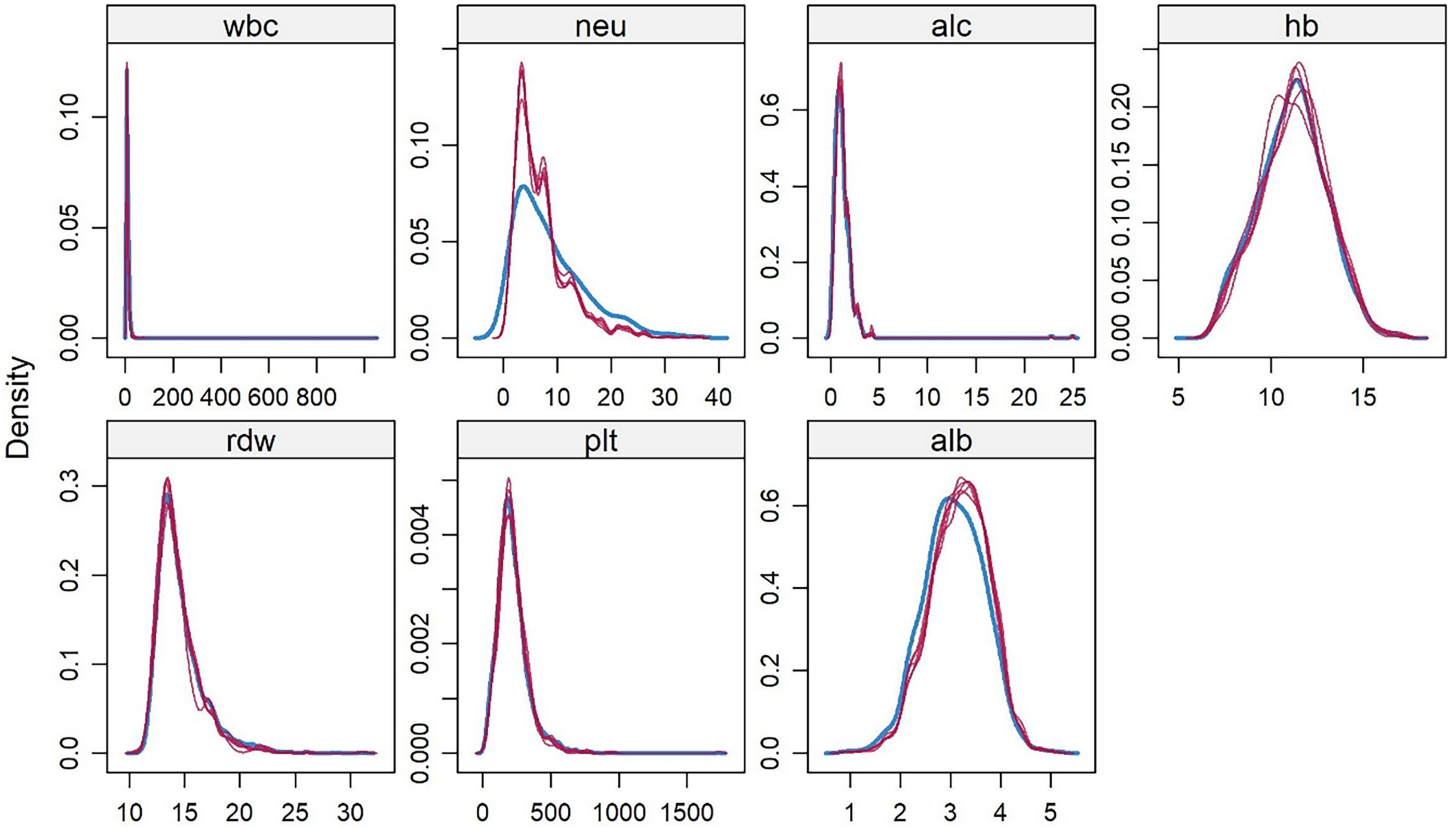
Kernel Density Distribution of Hematological Indicators in AP Patients from the MIMIC-IV 3.1 Database. White blood cell count (wbc), neutrophil count (neu), lymphocyte count (alc), hemoglobin (hb), red blood cell distribution width (rdw), platelet count (plt), albumin (alb). This figure visually shows that after 5 rounds of random forest imputation in the derivation cohort, the density of each indicator is highly consistent with the original complete data.

**FIGURE 2:**
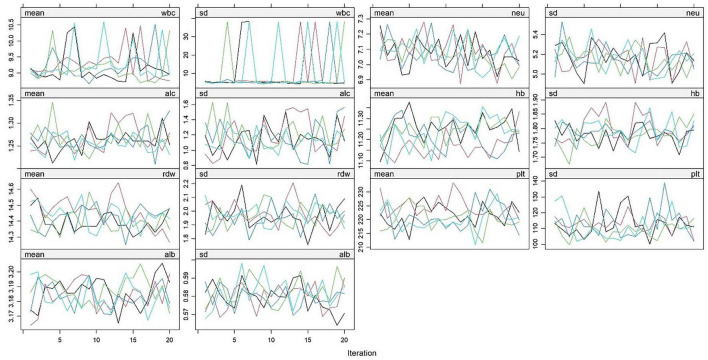
Changes in mean and standard deviation of hematological indicators in AP patients from the MIMIC-IV 3.1 database during random forest imputation iterations. White blood cell count (wbc), neutrophil count (neu), lymphocyte count (alc), hemoglobin (hb), red blood cell distribution width (rdw), platelet count (plt), albumin (alb). This figure reflects the changing trends of the mean (mean) and standard deviation (sd) of the above hematological indicators with the number of iterations. Colored lines represent indicator fluctuations in each imputed dataset, indicating stable convergence of the imputation process.

A derivation cohort was constructed based on clinical data of 4,930 AP patients from the MIMIC-IV 3.1 database (2008–2022). Given the high missing rates of multiple key variables, to avoid sample size reduction and information bias caused by complete case analysis, and to avoid underestimation of data variability by single-value imputation, this study used the random forest algorithm for multiple imputation. Data preprocessing was performed before imputation: (1) Clarify variable attributes and clinically reasonable ranges. Among them, L (lymphocyte count) and N (neutrophil count) were count data (unit: × 10^9^/L), and Alb (albumin) and Hb (hemoglobin) were continuous data. Extreme outliers were removed using the ±3 standard deviation method combined with clinical reference ranges (e.g., WBC count 4–10 × 10^9^/L); (2) Sort out variable correlations based on clinical logic (e.g., the compositional relationship between WBC count and neutrophil count, and the anemic correlation between Hb and RDW), and screen predictive variables for the imputation model. The imputation process was as follows: (1) The number of imputed datasets was set to 5 (referring to the classic recommended range of 3–10 for multiple imputation, balancing the reflection of missing value uncertainty and computational efficiency); (2) Construct a random forest imputation model: Using 48-h WBC count and 48-h hemoglobin with low missing rates as predictive variables, a separate model was built for each missing variable (e.g., 72-h lymphocyte count). 100 decision trees were generated through bootstrap sampling, and the missing value imputation results were output using the strategy of “mode voting for count data and mean integration for continuous data”; (3) Repeat the above steps until all missing values are filled, and finally obtain 5 complete datasets with consistent structure and reasonable differences in imputed values. The imputation effect was verified through multiple dimensions: (1) Density plots and histograms showed that the distribution of variables after imputation was highly consistent with the original complete data (Figure 1). (2) The convergence curves of variance and mean after multiple iterations of each variable showed no obvious divergence trend in the distribution of imputed data. Variables with obvious skewness were log-transformed to correct the skewness (Figure 2).

### 3.2 Multivariate logistic regression analysis of the derivation cohort

In the multivariate Logistic regression analysis after multiple imputation, several variables showed statistical significance: the odds ratio (OR) of log(NLR) was 2.92 (95% confidence interval 2.02–4.22, *p* < 0.001), indicating it was a significant risk-increasing factor; the OR of log(SII) was 0.36 (95% confidence interval 0.25–0.50, *p* < 0.001), which was a significant risk-reducing factor; the OR of log(WBC) was 1.83 (95% confidence interval 1.21–2.75, *p* = 0.009), suggesting an association with increased risk; hemoglobin (Hb) had an OR of 0.87 (95% confidence interval 0.82–0.92, *p* < 0.001), indicating it was a protective factor for risk reduction; red blood cell distribution width (RDW) had an OR of 1.13 (95% confidence interval 1.09–1.18, *p* < 0.001), suggesting a significant association with increased risk. Albumin (Alb, *p* = 0.165), lymphocyte count (L, *p* = 0.649), platelets (PLT, *p* = 0.157), and neutrophils (N, *p* = 0.136) showed no statistical significance (Table 1).

**TABLE 1:** Comparison of the clinical baseline data between the NSAP group and the SAP group.

Characteristic	OR	95% CI	*P*-value
log(NLR)	2.92	2.02, 4.22	<0.001*
log(SII)	0.36	0.25, 0.50	<0.001*
log(WBC)	1.83	1.21, 2.75	0.009*
Hb	0.87	0.82, 0.92	<0.001*
RDW	1.13	1.09, 1.18	<0.001*
Alb	0.84	0.66, 1.08	0.165
L	1.02	0.92, 1.14	0.649
PLT	1.00	1.00, 1.00	0.157
N	1.04	0.98, 1.10	0.136

CI, confidence interval; OR, odds ratio. *P < 0.05.

### 3.3 Nomogram model construction

**FIGURE 3:**
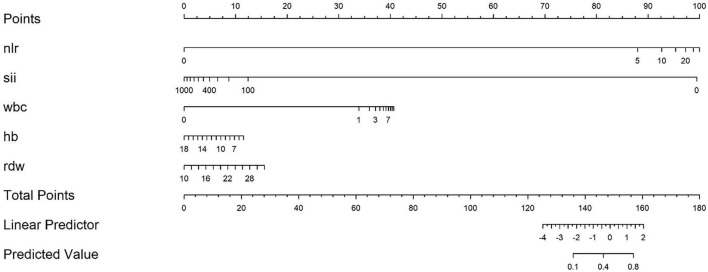
Nomogram model for predicting severe risk of acute pancreatitis. Based on the actual values of each indicator corresponding to the “Points” axis score, the SAP prediction probability is read through the “Total Points” axis after accumulation.

The nomogram Logistic regression model further screened and included 5 statistically significant variables, with an OR of log(NLR) of 2.37 (95% confidence interval 2.03–2.77, *p* < 0.001), log(SII) of 0.45 (95% confidence interval 0.39–0.53, *p* < 0.001), log(WBC) of 2.60 (95% confidence interval 2.18–3.10, *p* < 0.001), Hb of 0.85 (95% confidence interval 0.81–0.89, *p* < 0.001), and RDW of 1.14 (95% confidence interval 1.10–1.19, *p* < 0.001). All included variables showed stable statistical significance, indicating that the model retained key predictive factors after variable simplification (Table 2). A prediction model equation was established with 5 influencing factors: logit(P) = ln(2.37)⋅ log(NLR) + ln(0.45)⋅ log(SII) + ln(2.60)⋅ log(WBC) + ln(0.85)⋅ Hb + ln(1.14)⋅ RDW, and a nomogram model was constructed (Figure 3).

**TABLE 2:** Nomogram logistic regression.

Characteristic	OR	95% CI	*P*-value
log(NLR))	2.37	2.03, 2.77	<0.001*
log(SII)	0.45	0.39, 0.53	<0.001*
log(WBC))	2.60	2.18, 3.10	<0.001*
Hb	0.85	0.81, 0.89	<0.001*
RDW	1.14	1.10, 1.19	<0.001*

CI, confidence interval; OR, odds ratio. *P < 0.05.

### 3.4 Model evaluation of modeling queue

Overall, the model constructed in this study selected inflammation and hematological indicators with significant predictive value by handling missing values reasonably. The model performed well in discrimination and calibration, providing reliable statistical basis for risk prediction of related outcome events. The detailed description is as follows:

#### 3.4.1 ROC curve of modeling queue

**FIGURE 4:**
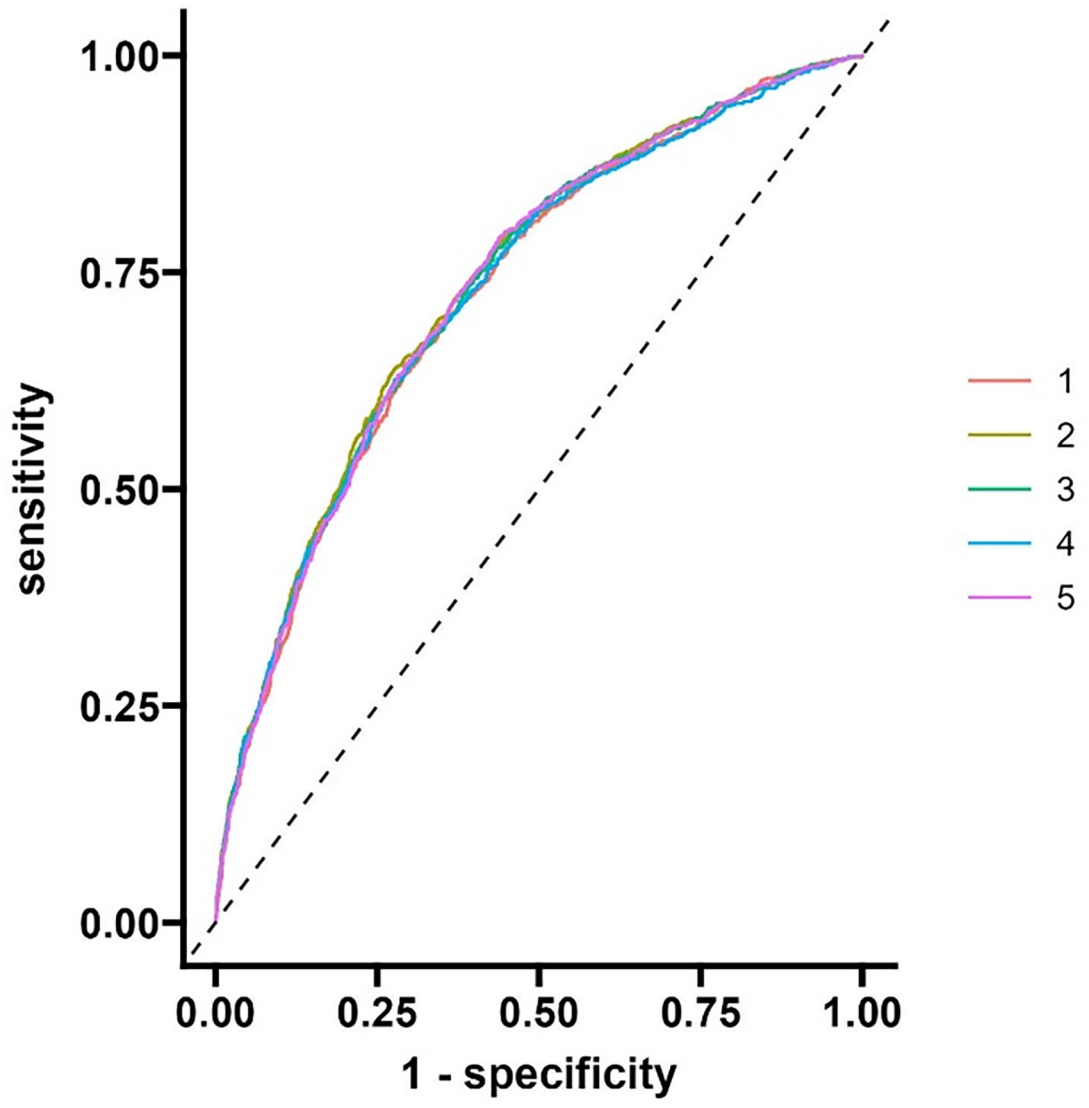
ROC curve of modeling queue nomogram model.

The evaluation results of the model show that the area under the curve (AUC) of the discrimination index calculated using the Rubin rule after multiple interpolation modeling is 0.730 (95% confidence interval 0.710–0.749), indicating that the model has a medium to high discriminatory ability and can effectively distinguish the risk of different outcome events (Figure 4 and Table 3).

**TABLE 3:** Nomogram logistic regression.

Rowname	95% Low	C-statistic	95% Up
AUC	0.710	0.730	0.749

#### 3.4.2 Calibration of modeling queue models

**FIGURE 5:**
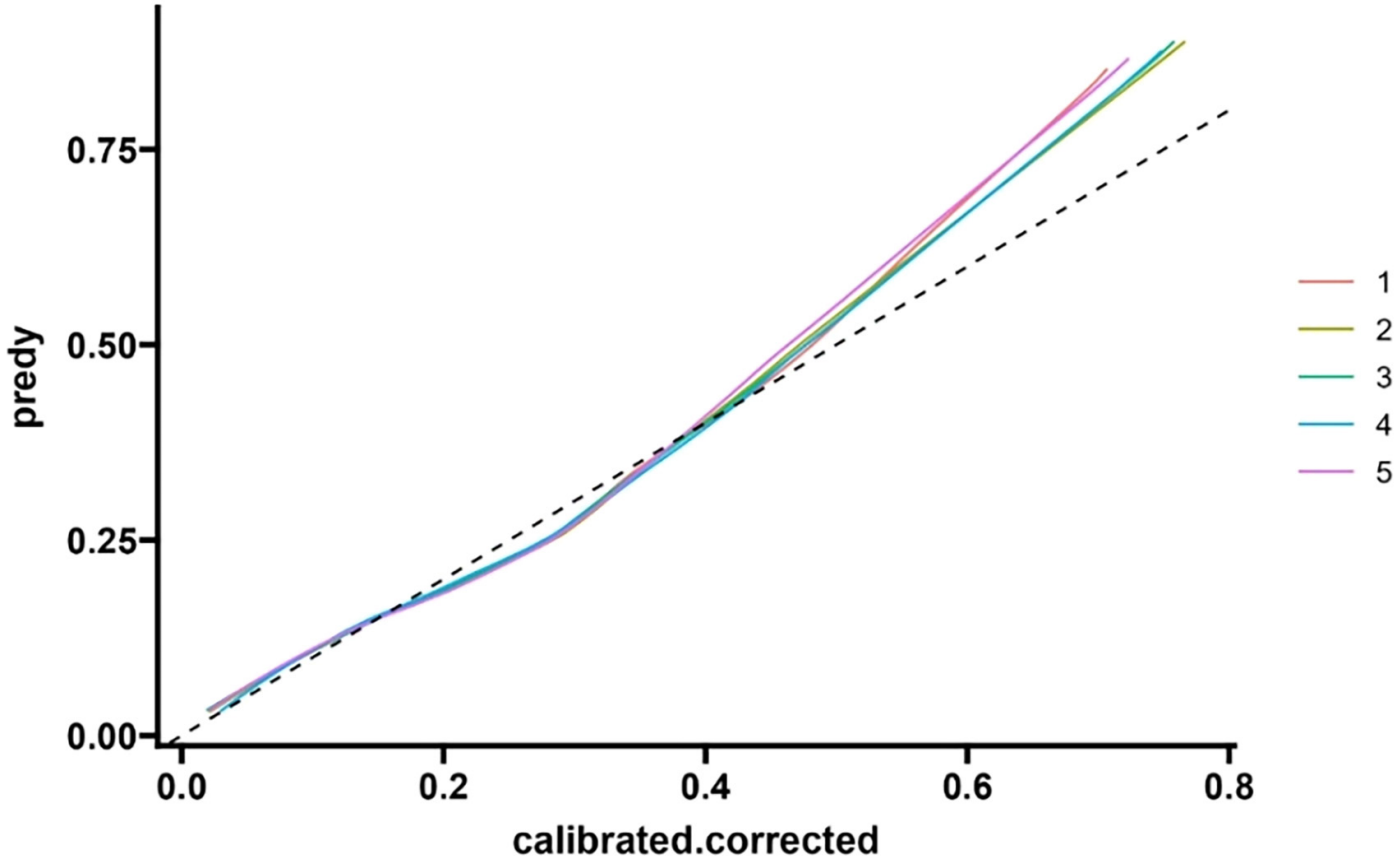
Modeling queue nomogram model calibration curve.

The *F*-value of the Hosmer-Lemeshow test (HL test) for the calibration index is 1.320, with a *p*-value of 0.333 (degree of freedom 1 = 8.000, degree of freedom 2 = 10.176). As the *p*-value is greater than 0.05, it indicates that the model’s predicted probability has good consistency with the actual observation results, and the calibration effect is ideal. See Figure 5 and Table 4.

**TABLE 4:** Nomogram logistic regression.

Rowname	*F*-value	*P*(>*F*)	df1	df2
HL test	1.320	0.333	8.000	10.176

#### 3.4.3 Decision analysis curve of modeling queue

**FIGURE 6:**
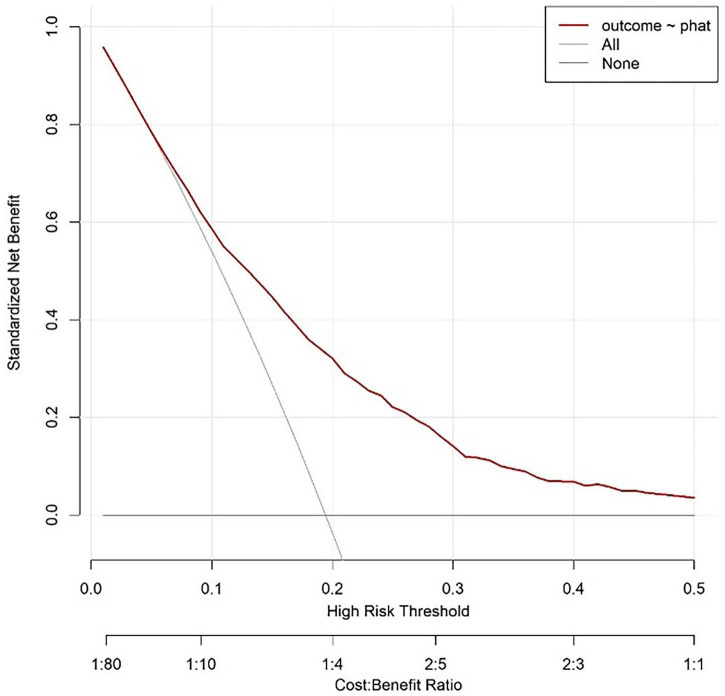
Modeling queue nomogram model decision analysis curve.

The curve corresponding to this research model is located above the “all intervention” and “no intervention” curves, indicating that using this model for risk prediction and guiding clinical decision-making within this interval can achieve net benefits higher than extreme strategies (i.e., intervention or no intervention for all individuals). Based on the labeled Cost Benefit Ratio (such as 1:80–1:1) in the figure, when the risk threshold matches a specific cost-benefit ratio, the net benefit advantage of the model is more significant, indicating that in the corresponding clinical scenarios, the model can effectively help screen out individuals who truly need intervention, reduce unnecessary medical resource consumption or missed diagnosis risks, and has good clinical practicality (Figure 6).

### 3.5 Validation of the column chart model for the validation queue

**FIGURE 7:**
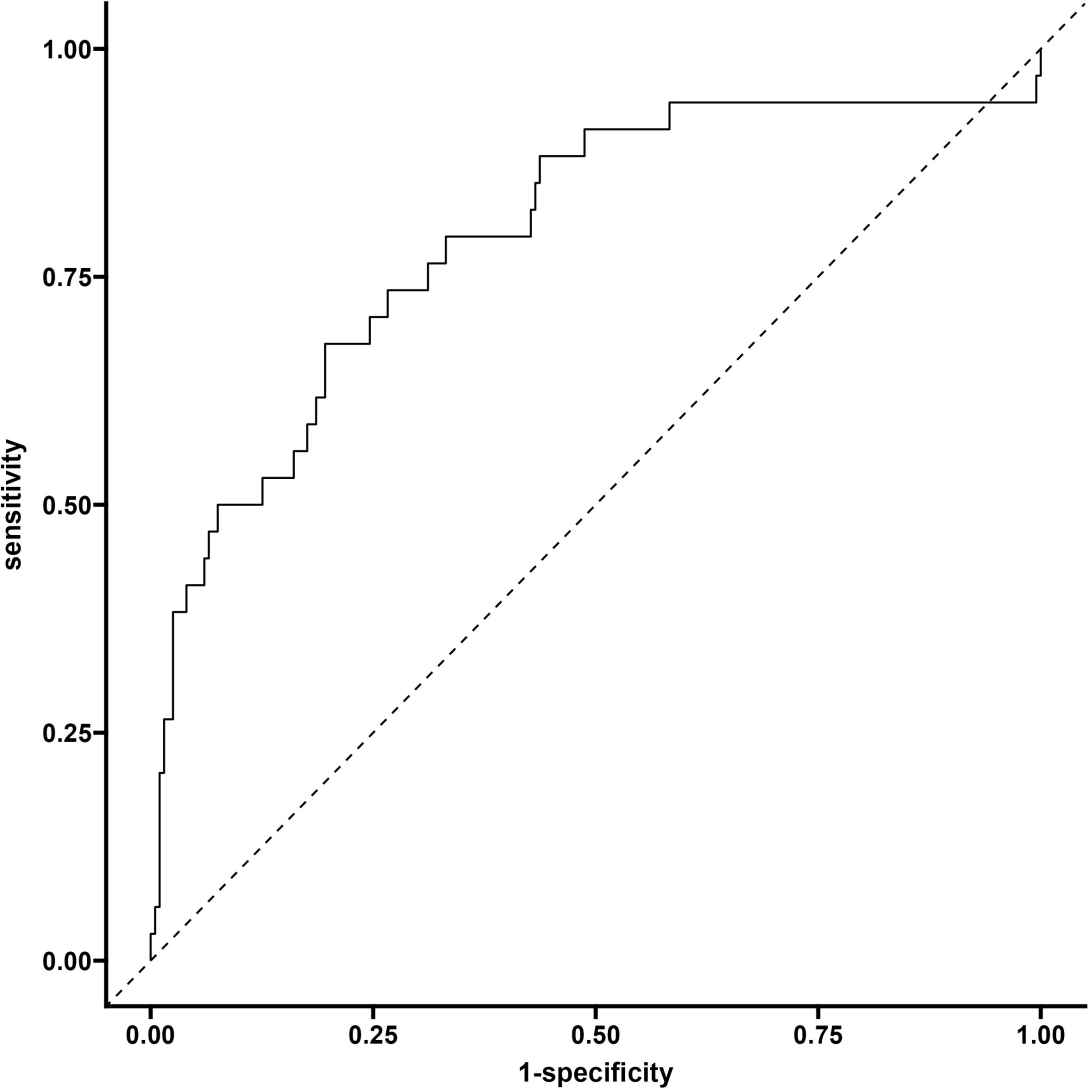
Validate the ROC curve of the queue.

The model evaluation results showed that the area under the curve (AUC) of the discrimination index calculated using the Rubin rule after multiple interpolation modeling was 0.795 (95% CI: 0.703–0.886), indicating that the model has a medium to high discrimination ability and can effectively distinguish the risk of different outcome events (Figure 7).

**FIGURE 8:**
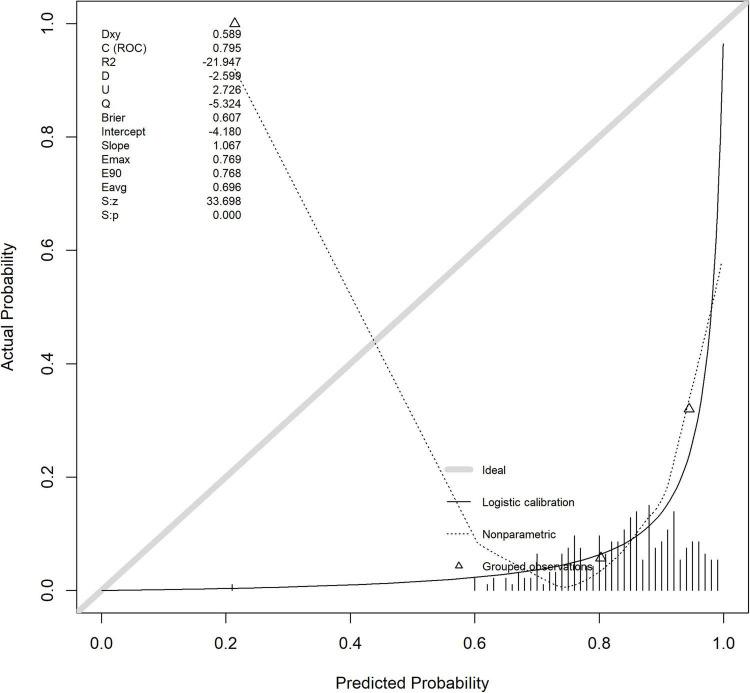
Verify the calibration curve of the queue.

The horizontal axis of the calibration curve, labeled as “Ideal”, may deviate from the actual calibration curve due to the presence of extreme risk value samples (such as a very small number of extremely high-risk or low-risk individuals) or insufficient sample size in a certain risk interval, which may result in inaccurate probability estimation of the model in that interval. The calibration index Hosmer Lemeshow test (HL test) has a chi square value of 1.320 and a *p*-value of 0.333 (degrees of freedom = 8). As the *p*-value is greater than 0.05, it indicates that the model’s predicted probability is consistent with the actual observation results, and the calibration effect is ideal (Figure 8).

**FIGURE 9:**
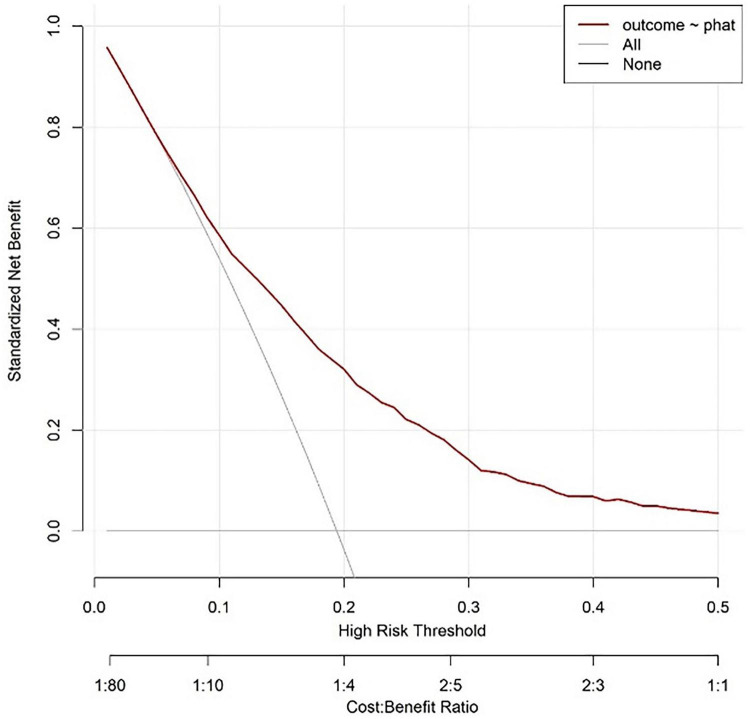
Validate the decision analysis curve of the queue.

The horizontal axis of the decision curve represents the high-risk threshold or cost-benefit ratio, and the vertical axis represents the net benefit (0.00–0.80). The decision curve corresponding to the model is higher than the “All” and “None” lines in most threshold ranges. Based on this model, setting a high-risk threshold for intervention results in better net benefits, which can support clinical selection of reasonable prediction thresholds to balance intervention benefits and costs, and has clinical application value (Figure 9).

## 4 Discussion

The core challenge in clinical management of acute pancreatitis (AP) lies in early identification of severe subtypes (SAP), which are often accompanied by a vicious cycle of persistent inflammation, immune suppression, and high catabolic metabolism (i.e., persistent inflammation immune suppression catabolic syndrome, PICS) (6), with a mortality rate of up to 20%, while non severe AP (NSAP) is often self-limiting (1). This study focuses on the critical window period of 48–72 h after the onset of acute pancreatitis (AP), which is a clinical node for the transformation of systemic inflammatory response syndrome (SIRS) to PICS (17). Fluid imbalance is basically corrected, and hematological indicators have more reference value (18). Based on this, we constructed and validated a Nomogram model based on blood routine indicators, aiming to provide a simple and efficient tool for SAP risk prediction. The following is a comprehensive analysis based on research results, existing evidence, and clinical needs.

This study conducted large-scale modeling (4930 cases in MIMIC-IV database) and single center external validation (233 cases), and found that NLR, SII, WBC, Hb, and RDW were independent predictive factors for SAP. The Nomogram model constructed based on this showed good predictive performance: the AUC of the modeling queue reached 0.730 (95% CI: 0.710–0.749, above average discriminative ability), and the calibration test Hosmer Lemeshow (HL) *p* = 0.333 (good consistency between prediction and actual results); The net benefit analysis shows that the model outperforms the extreme strategies of “all intervention” and “no intervention” (with a cost-effectiveness ratio range of 1:80–1:1), indicating that the model can effectively help screen out individuals who truly need intervention, reduce unnecessary medical resource consumption or the risk of missed diagnosis, and has good clinical practicality. The validation queue AUC was further increased to 0.795 (95% CI: 0.703–0.886), and the model still maintained good predictive performance, indicating its potential application value in clinical practice.

NLR (neutrophil/lymphocyte ratio) and SII (platelet × NLR) are the core inflammatory immune markers of the model. The elevation of NLR is essentially an imbalance between “neutrophil pro-inflammatory activation” and “lymphocyte immune exhaustion” (19). In the progression of acute pancreatitis, inflammatory factors such as IL-6 and TNF-α drive neutrophil aggregation and release extracellular traps (NETs), exacerbating tissue damage, while inducing lymphocyte apoptosis (especially CD4^+^ T cells), leading to immune suppression (20). SII is incorporated into platelets on the basis of NLR, further reflecting the coagulation disorder in PICS. During SAP, pancreatic necrosis activates the coagulation system, and a large number of platelets are consumed for microthrombus formation. Therefore, changes in SII values are positively correlated with the degree of immune suppression (19, 21). In this study, the OR of log (NLR) was 2.37 (*p* < 0.001) and the OR of log (SII) was 0.45 (*p* < 0.001), indicating that both capture the inflammation immune imbalance of PICS through different dimensions and jointly improve the prediction accuracy of the model.

The PICS characteristics of SAP not only include inflammation immune abnormalities, but also continuous catabolism. Hb and RDW in blood routine are good indicators reflecting this state (22). Hormonal disorders under stress (such as elevated cortisol) promote protein breakdown and gluconeogenesis, leading to reduced hemoglobin synthesis and insufficient raw materials for red blood cell production, manifested as decreased Hb; At the same time, inflammation interferes with iron metabolism, leading to premature release of immature red blood cells and causing uneven red blood cell size, i.e., an increase in RDW (23). Although traditional indicators such as Alb are the most commonly used nutritional evaluation markers, they also have important value in evaluating AP (24). However, Ocskay et al.’s large sample study showed that only 19% of AP patients had hypoalbuminemia upon admission, and 25% of patients only experienced Alb decline in the middle and later stages of hospitalization, with a significant lag (25). This is consistent with the results of our study. We found that in the early stages of admission, Alb decline in AP patients was not significant (*p* = 0.165), so it did not enter the final model. Hb and RDW, as indicators that can be obtained within 48–72 h of admission, can reflect the nutritional consumption characteristics of PICS earlier, which also explains why the model performs better in early prediction. Elevated WBC is a direct sign of persistent inflammation in AP, and its numerical changes are mainly driven by neutrophils, which are positively correlated with the extent of pancreatic necrosis and the risk of organ failure. In this study, the OR of log (WBC) was 2.60 (*p* < 0.001), further confirming the clinical consensus that “basal inflammatory intensity is a prerequisite for the occurrence of SAP.”

The current SAP prediction tools mainly have the following limitations: traditional scoring systems have disadvantages such as complex operation and strong lag (7, 26), such as APACHE II requiring more than 20 indicators, MCTSI relying on enhanced CT 48 h after onset, and early pancreatic necrosis not fully manifested. Various single center studies often have poor credibility due to small sample sizes and selection biases, and are often based solely on inflammation indicators or a combination of nutritional indicators.

The characteristic of this study lies in integrating the three dimensions of “inflammation (WBC, NLR) - immunity (SII) - nutrition (Hb, RDW)” based on the PICS theory, breaking through the limitations of “predicting solely based on inflammation indicators”, and better fitting the pathophysiological essence of SAP (27). The modeling queue uses the MIMIC-IV database with 4930 large samples (covering the population from 2008 to 2022) to avoid overfitting caused by small samples; The independent validation queue included 233 patients in our hospital from 2016 to 2024, achieving external validation across databases and time periods, in compliance with international standards for predictive model construction (28). In addition, the indicators included in this study have higher practicality: all predictive indicators are derived from routine blood routine tests (without the need for special equipment or delayed examinations), which can be quickly obtained in emergency or primary hospitals, solving the pain points of “complex operation and equipment dependence” in traditional scoring. Compared with similar studies, this model only relies on blood routine indicators and has more clinical value in balancing convenience and efficacy. Therefore, we hope that through this study, we can provide a “practical risk stratification tool” for clinical doctors to intervene early in SAP. Within 48–72 h of admission for AP patients, doctors can quickly calculate Nomogram scores based on blood routine indicators. For high-risk patients (such as those with a predicted probability >45%), priority should be given to initiating enhanced monitoring (such as continuous SOFA scores, dynamic follow-up of blood routine and biochemical indicators) and intervention measures (such as early enteral nutrition and use of immunomodulators) to break the vicious cycle of “inflammation → immunosuppression → nutritional depletion” in PICS; For low-risk patients, excessive medical treatment (such as unnecessary enhanced CT or ICU transfers) should be avoided to reduce waste of medical resources. The advantage of Nomogram is that it presents risks in a visual chart (Figure 3), without the need for complex calculations. Doctors only need to correspond scores based on the actual values of patient indicators such as NLR and SII, and accumulate them to read SAP risk probability. It is particularly suitable for emergency busy scenes or primary hospitals. The uniqueness of this study lies in strengthening awareness of nutritional intervention: the inclusion of Hb and RDW in the model suggests that “nutritional assessment should be integrated into early management of AP”. In clinical practice, these two indicators can be used to identify high-risk patients for nutritional depletion in advance, so as to initiate enteral nutrition support earlier and avoid severe hypoalbuminemia or infection complications in the middle and later stages (24).

Although this study strives for rigor in design and analysis, there are still the following shortcomings that need to be objectively addressed:

The modeling queue is sourced from the MIMIC-IV database (mainly for European and American populations), while the validation queue is only single center data from the Anhui No.2 Provincial People’s Hospital, which may not fully represent AP patients in other regions of Asia or different medical systems [such as differences in etiology composition: alcohol induced AP is more common in European and American populations, and biliary AP is more common in Asian populations (29)]. The extrapolation of the model requires further validation through multi center and cross ethnic studies. In addition, the study did not include potential confounding factors such as the etiology of AP (such as biliary, alcoholic, hyperlipidemic), treatment regimen (such as fluid resuscitation volume, antibiotic use), etc. There are differences in the incidence of SAP among different etiologies (such as hyperlipidemia induced AP being more likely to progress to severe cases (30, 31), which may affect the accuracy of model prediction). In addition, inflammatory markers such as CRP and PCT actually have great value in the assessment of the condition of AP patients (32). However, this study did not include them due to the high missing rate of the MIMIC-IV database. In the future, their optimization value for the model can be supplemented and evaluated in a complete data queue. Limitations of retrospective design: This study is a retrospective analysis, and although missing values were processed using random forest interpolation (20 iterations, generating 5 datasets), data recording bias could not be completely avoided; Moreover, retrospective design cannot verify whether model-based interventions improve prognosis, and prospective intervention studies are needed to further confirm the clinical benefits of the model.

Based on the above limitations, future multicenter prospective cohort studies need to be conducted, incorporating variables such as the etiology of AP, imaging features (such as pancreatic necrosis range), and treatment measures, to construct a multidimensional model of “hematology + clinical + imaging”; Simultaneously developing stratified models for different subgroups of etiology (such as biliary and alcoholic) to enhance predictive specificity. If conditions permit, randomized controlled trials can be conducted to compare the prognostic differences (such as SAP incidence, length of hospital stay, mortality) between “Nomogram based risk stratification intervention” and “conventional diagnosis and treatment”, and to clarify the application value of the model in practical clinical scenarios.

## 5 Conclusion

This study is based on the PICS theory and constructs and validates an SAP prediction Nomogram model with NLR, SII, WBC, Hb, and RDW as the core. The model has the advantages of easy access to indicators, reliable predictive performance, and high clinical usability, and can provide a practical tool for early identification of SAP during the critical window period of 48–72 h after the onset of AP. Despite the limitations of population bias and retrospective design, it lays the foundation for precise risk stratification and early intervention of AP. In the future, through multi center optimization and prospective validation, it is expected to become a routine auxiliary tool for clinical management of AP.

## Data Availability

The raw data supporting the conclusions of this article will be made available by the authors, without undue reservation.
